# Association of probiotics combined with glutamine with postoperative outcomes in children with Hirschsprung disease: a retrospective propensity score-matched cohort study

**DOI:** 10.3389/fped.2026.1953553

**Published:** 2026-10-05

**Authors:** Peiyu Zhao, Qingqing Wang, Fang Wei, Guoqi Zheng, Shanshan Su

**Affiliations:** 1 Department of Gastrointestinal Surgery/Pediatric Surgery, Renmin Hospital, Hubei University of Medicine, Shiyan City, Hubei, China; 2 Digestive Department, Cangzhou Central Hospital, Cangzhou City, Hebei, China

**Keywords:** children, enterocolitis, glutamine, Hirschsprung disease, postoperative, probiotics

## Abstract

**Objective:**

To evaluate the association between postoperative use of probiotics combined with glutamine and intestinal function recovery, Hirschsprung disease-associated enterocolitis (HAEC), nutritional status, and immune-inflammatory indicators following radical surgery for Hirschsprung disease (HD).

**Methods:**

This retrospective observational cohort study included children who underwent radical surgery for HD at our hospital between January 2023 and December 2025. According to the actual postoperative regimen received, patients were divided into an observation group receiving probiotics combined with glutamine in addition to conventional postoperative management (*n* = 46) and a control group receiving conventional postoperative management alone without the probiotic preparation or glutamine supplementation used in the observation group (*n* = 48). A 1:1 propensity score matching (PSM) was performed to reduce confounding from measured baseline characteristics and improve covariate balance between the non-randomized treatment groups, resulting in 40 matched pairs. Postoperative recovery of bowel sounds, time to first defecation, length of hospital stay, HAEC incidence at 6 months postoperatively, and changes in nutritional markers (albumin, prealbumin, transferrin), peripheral blood T lymphocyte subsets (CD4⁺, CD4⁺/CD8⁺ ratio), and inflammatory cytokines (IL-10, IL-6) were compared between the two groups.

**Results:**

After PSM, the observation group exhibited significantly shorter times for recovery of bowel sounds, first defecation, and hospital stay compared to the control group [(22.5 ± 7.8) h vs. (34.6 ± 9.5) h, (38.5 ± 10.1) h vs. (51.8 ± 12.5) h, (10.5 ± 3.0) d vs. (14.4 ± 4.2) d, all *P* < 0.001]. At 6 months postoperatively, the incidence of HAEC was lower in the observation group than in the control group [10.0% (4/40) vs. 32.5% (13/40), *P* = 0.014]. Baseline nutritional, inflammatory, and cellular immune indicators were comparable between the matched groups (all *P* > 0.05). From baseline to 3 months, the observation group showed greater increases in albumin, prealbumin, transferrin, IL-10, CD4 percentage, and CD4/CD8 ratio and a greater decrease in IL-6 than the control group (all *P* < 0.001).

**Conclusion:**

In this retrospective observational cohort, postoperative use of probiotics combined with glutamine was associated with earlier intestinal function recovery, shorter hospitalization, a lower incidence of HAEC, and more favorable nutritional, cellular immune, and inflammatory indicators after PSM. These findings should be interpreted as associations rather than evidence of a causal treatment effect and require confirmation in prospective randomized studies.

## 1 Introduction

Hirschsprung disease (HD) is a relatively common congenital gastrointestinal malformation in pediatrics, characterized pathologically by the absence of ganglion cells in the distal bowel segment, leading to sustained intestinal spasm and functional obstruction (1, 2). Radical pull-through surgery remains the only effective curative treatment; however, postoperative patients frequently encounter clinical challenges such as delayed intestinal function recovery, malnutrition, and Hirschsprung disease-associated enterocolitis (HAEC). HAEC is the most severe and potentially life-threatening complication following HD surgery, with onset possible at any time postoperatively. Current evidence suggests that its pathogenesis is closely associated with three key factors: gut microbiota dysbiosis, intestinal mucosal barrier injury, and local immune-inflammatory dysregulation (3–5).

Surgical trauma from HD resection, combined with routine perioperative fasting and antibiotic use, often exacerbates gut microbiota imbalance and intestinal mucosal atrophy. Glutamine, as a critical energy substrate for intestinal mucosal cells, helps maintain barrier integrity during stress and reduces bacterial translocation. Probiotics, through multiple mechanisms—including modulation of gut microbial balance, competitive inhibition of pathogen colonization, and immune regulation—provide protective effects on the intestinal tract. Most prior studies have focused on single-intervention strategies, and evidence regarding the combined postoperative use of probiotics and glutamine remains limited. Therefore, this retrospective observational cohort study used propensity score matching to evaluate the association between postoperative probiotics combined with glutamine and intestinal function recovery, HAEC incidence, and subsequent nutritional, immune, and inflammatory indicators in children with HD, with the aim of providing observational evidence for further evaluation of this postoperative management strategy.

## 2 Materials and methods

### 2.1 Study subjects

This study adopted a single-center retrospective observational cohort design, with data collection and analysis conducted in accordance with the STROBE statement. Study subjects were identified from medical records of pediatric patients who underwent radical HD surgery in the Department of General Surgery of our hospital between January 2023 and December 2025. The radical pull-through procedures performed during the study period comprised laparoscopic-assisted pull-through and transanal pull-through.

The study protocol was reviewed and approved by the Ethics Committee of Cangzhou Central Hospital (Approval No. 23DX-EKLCYY01). The requirement for written informed consent was waived by the Ethics Committee because of the retrospective nature of the study and the use of existing clinical records. All data were de-identified before analysis, and the study was conducted in accordance with the Declaration of Helsinki and relevant institutional requirements. The patient screening, exclusion, grouping, and propensity score matching process are shown in Figure 1.

**Figure 1:**
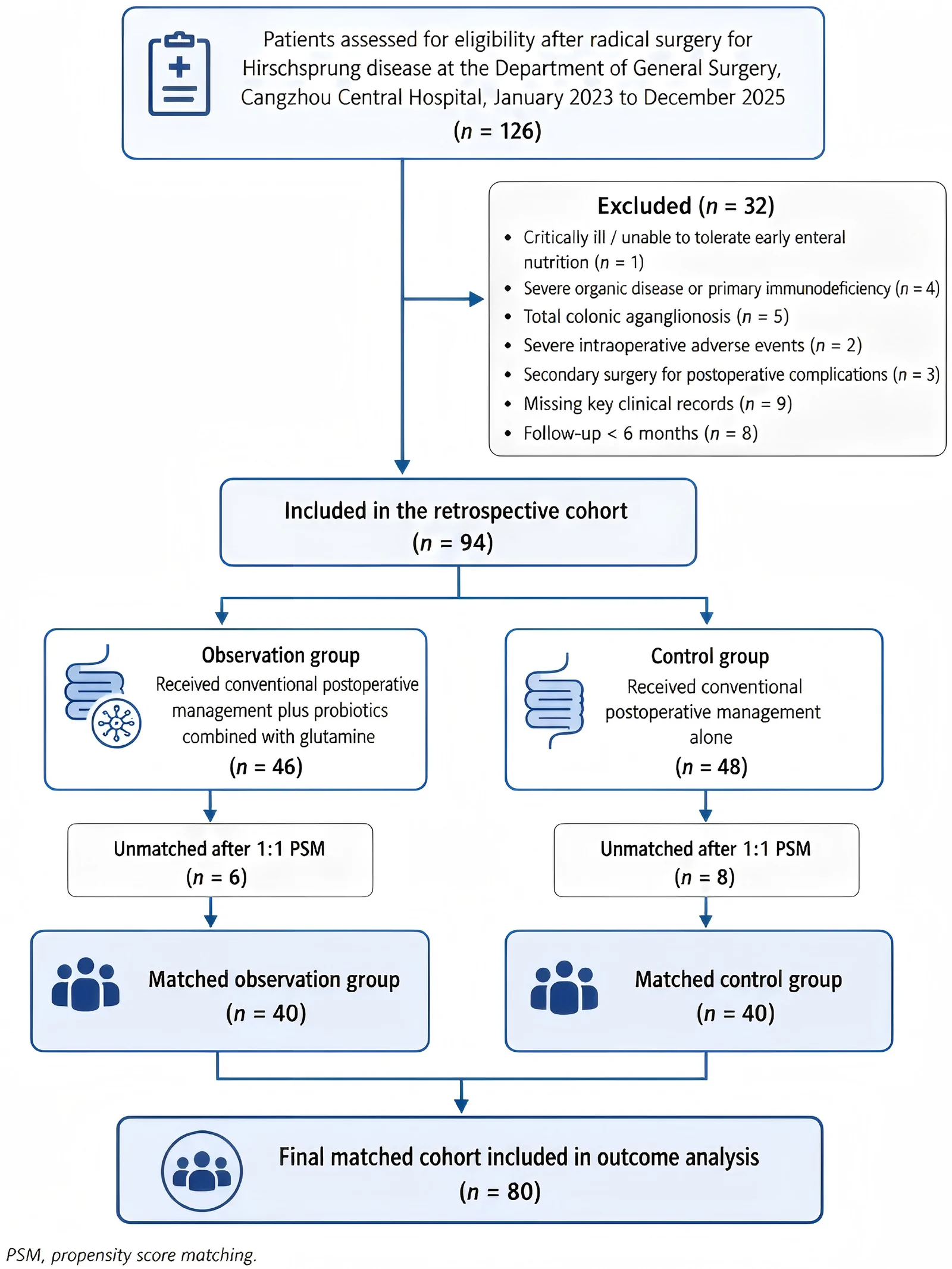
Flow diagram of patient screening, exclusion, grouping, and propensity score matching.

A total of 126 children who underwent radical surgery for Hirschsprung disease between January 2023 and December 2025 were screened. After exclusion of 32 patients who did not meet the eligibility criteria, 94 patients were included in the retrospective cohort, including 46 in the observation group and 48 in the control group. Following 1:1 propensity score matching (PSM), 40 matched pairs (*n* = 80) were included in the final analysis.

### 2.2 Inclusion and exclusion criteria

Complete clinical diagnosis and treatment records, as well as postoperative follow-up data for all enrolled subjects, were retrieved from the hospital's electronic medical record system. The screening process was strictly implemented following pre-established inclusion and exclusion criteria.

Inclusion criteria: Children with a definitive diagnosis of Hirschsprung disease confirmed by routine postoperative pathological sections (6–8); aged under 6 years who received their first radical pull-through surgery; with stable postoperative vital signs and hemodynamic parameters, and able to successfully initiate enteral nutrition support within 48 h after surgery.

Exclusion criteria: Critically ill cases intolerant to early enteral nutrition; children with pre-existing severe organic diseases of the heart, liver or kidney, or primary immunodeficiency; cases pathologically classified as total colonic aganglionosis, or those with severe intraoperative adverse events such as intestinal perforation; children who underwent secondary surgery for reasons including postoperative anastomotic stricture or intestinal obstruction; cases with missing key clinical diagnosis and treatment records, or postoperative follow-up duration less than 6 months. Total colonic aganglionosis was excluded because it represents a clinically distinct and more extensive form of HD that generally requires different surgical and postoperative management and may be associated with substantially different nutritional requirements, bowel adaptation, and postoperative functional outcomes. Its inclusion could therefore have introduced considerable clinical heterogeneity and confounding when evaluating postoperative bowel recovery and nutritional outcomes in the present cohort.

A total of 94 children meeting all the criteria were finally enrolled in the study, and were grouped according to the actual postoperative intervention regimen they received.

### 2.3 Intervention regimen

The basic postoperative management for children in both groups followed the same unified clinical pathway after radical surgery for Hirschsprung disease. Accordingly, routine postoperative anti-infective treatment, nutritional support, progression of enteral feeding, correction of fluid and electrolyte disturbances, and discharge assessment were managed according to the same institutional postoperative pathway in both groups. The control group received conventional postoperative management alone and did not receive the probiotic preparation or L-glutamine capsules used in the observation group during the 1-month intervention period. Conventional postoperative management included preventive anti-infective treatment, immunomodulatory support, individualized nutritional supplementation, and symptomatic treatment, including dynamic correction of water and electrolyte disturbances according to the children's biochemical findings.

On the basis of the same conventional postoperative management, the observation group additionally received probiotics combined with glutamine. The probiotic preparation was Live Combined Bifidobacterium and Lactobacillus Tablets (Jinshuangqi®, Inner Mongolia Shuangqi Pharmaceutical Co., Ltd., China; 0.5 g/tablet; NMPA approval No. S19980004). Each tablet contained viable Bifidobacterium longum at not less than 0.5 × 10^7^ CFU, Lactobacillus delbrueckii subsp. bulgaricus (Lactobacillus bulgaricus) at not less than 0.5 × 10^6^ CFU, and Streptococcus thermophilus at not less than 0.5 × 10^6^ CFU. Specific strain accession numbers were not available in the retrospective medication records; therefore, the labeled species composition is reported. Children aged ≥3 years received 3 tablets orally three times daily, whereas children aged <3 years received 2 tablets orally twice daily. When systemic antibiotics were administered concurrently, the probiotic and antibiotic doses were separated by at least 2 h. Glutamine was administered as L-glutamine capsules (Ziwei®, Jiangsu Shenhua Pharmaceutical Co., Ltd., China; 0.25 g/capsule; NMPA approval No. H20010192), at a dose of 3 capsules (0.75 g) orally three times daily, corresponding to a total daily dose of 2.25 g. The glutamine regimen was prescribed as a fixed dose rather than on a body-weight basis; therefore, a protocol-defined dose in g/kg/day was not available. Based on the mean body weight of approximately 8.65 kg in the matched cohort, the nominal average daily exposure was approximately 0.26 g/kg/day. Both probiotics and glutamine were initiated after enteral feeding had been tolerated, generally within 48 h after surgery, and were continued for 30 consecutive days.

### 2.4 Observation indicators

Perioperative variables potentially reflecting surgical complexity were also extracted from the operative and anesthesia records, including operative time (min) and estimated intraoperative blood loss (mL). Operative time was defined as the interval from skin incision to completion of wound closure. Primary indicators: The first part focused on outcomes related to postoperative intestinal function recovery, specifically recording the time when bowel sounds were first detectable after surgery, the time of the first spontaneous defecation, and the corresponding total postoperative hospital stay. The second primary outcome was the occurrence of Hirschsprung disease-associated enterocolitis (HAEC) during the 6-month postoperative follow-up period. A validated numerical HAEC scoring system was not routinely documented in the original clinical records; therefore, a numerical score was not reconstructed retrospectively. Instead, suspected HAEC episodes were identified using a predefined combination of clinical, radiographic, and laboratory findings, including diarrhea or watery stool, fever, abdominal distension, radiographic evidence of ileus or bowel dilatation, inflammatory cells in stool examination, and an increased peripheral white blood cell count (9, 10). All suspected HAEC episodes were independently reviewed by two pediatric surgeons using de-identified clinical records. Information identifying whether the patient had received probiotics combined with glutamine was masked during retrospective adjudication. Disagreements were resolved by consensus with a senior pediatric surgeon. HAEC events during the 6-month follow-up were identified from postoperative readmission records, outpatient and emergency-department records, and available follow-up documentation. Mild outpatient episodes were included when sufficient clinical information was available to satisfy the same predefined criteria. Episodes reported only by caregivers without adequate supporting clinical documentation were not classified as confirmed HAEC. Recurrent episodes were recorded during follow-up; however, for the primary patient-level incidence analysis, each patient was counted once if at least one confirmed HAEC episode occurred.

Secondary indicators were assessed at baseline and at the routine 3-month postoperative follow-up. Baseline values were defined as the most recent available measurements obtained within 48 h before surgery. The assessed indicators covered three dimensions: nutritional indicators, including serum albumin, prealbumin, and transferrin; peripheral blood T-lymphocyte indicators, including the CD4 percentage and CD4/CD8 ratio; and inflammatory indicators, including interleukin-10 (IL-10) and interleukin-6 (IL-6). For each indicator, the change from baseline (*Δ*) was calculated as the 3-month value minus the corresponding baseline value. Baseline and 3-month measurements were obtained using the same laboratory methods within each patient.

Treatment adherence and safety information were additionally extracted from inpatient medication administration records, discharge prescriptions, and follow-up records. Adherence to probiotics and glutamine was considered adequate when at least 80% of the prescribed doses during the 30-day intervention period were documented as administered or taken. Permanent discontinuation of the study regimen was considered when suspected drug allergy, persistent vomiting or diarrhea, inability to tolerate oral medication, serious clinical deterioration requiring interruption of enteral treatment, or a physician-determined safety concern occurred. Potential intervention-related adverse events, including gastrointestinal intolerance and allergic reactions, as well as any dose interruption or permanent discontinuation, were recorded during the treatment period.

### 2.5 Statistical methods

SPSS 26.0 software was used for statistical analysis. Because treatment allocation was non-random in this retrospective observational cohort, propensity score matching (PSM) was performed to reduce confounding from measured baseline characteristics and improve comparability between the two treatment groups. The absence of statistically significant differences in baseline characteristics was not considered sufficient evidence of covariate balance because significance testing is influenced by sample size. Receipt of the combined intervention was treated as the dependent variable. Baseline covariates available before initiation of the postoperative study intervention were selected for the propensity score model, including age at surgery (months), gender, body weight, disease segment classification, surgical approach, preoperative albumin, and preoperative antibiotic use time. Age was modeled as a continuous variable, and disease segment classification was used to account for differences in the anatomical extent of aganglionosis. These variables were selected because they could plausibly influence postoperative recovery or treatment selection and were consistently available in the retrospective records. Postoperative complications, postoperative antibiotic treatment, subsequent nutritional supplementation, enteral feeding progression, and hospital discharge were not entered into the baseline propensity score model because these variables occurred after treatment-group classification and could themselves be influenced by postoperative management or recovery. All 94 patients included in the final cohort had complete data for the variables entered into the propensity score model; therefore, complete-case analysis was used and no missing-data imputation was required. Patients were matched using 1:1 nearest-neighbor matching without replacement, with a caliper width of 0.02 on the propensity-score scale. The caliper of 0.02 was selected as a conservative matching threshold to restrict matches to patients with closely similar estimated probabilities of receiving the combined intervention while retaining an adequate number of matched pairs for analysis. Covariate balance before and after matching was assessed using standardized mean differences (SMDs), with an absolute SMD <0.10 considered indicative of acceptable balance. Baseline *P* values were reported descriptively and were not used as the primary criterion for determining covariate balance. Operative time and intraoperative blood loss were additionally compared between the two groups before and after matching as measured indicators of surgical complexity. No formal sensitivity analyses using alternative caliper widths, alternative matching algorithms, or propensity-score weighting methods were performed. Normally distributed continuous variables were expressed as mean ± standard deviation and compared using the independent-samples t-test; categorical variables were expressed as *n* (%) and compared using the *χ*^2^ test. For nutritional, inflammatory, and cellular immune indicators, change scores (*Δ* = 3-month value−baseline value) were calculated for each patient and compared between the two matched groups using the independent-samples t-test. Baseline values were also compared between groups to assess pre-intervention comparability. For continuous outcomes, between-group mean differences (MDs) with 95% confidence intervals (CIs) were additionally calculated as effect estimates. For the 6-month HAEC outcome, a post-matching univariable Logistic regression analysis was additionally performed with HAEC occurrence as the dependent variable and treatment group as the independent variable, and the odds ratio (OR) with 95% CI was reported. Because only 17 HAEC events occurred in the matched cohort, no additional covariates were entered into this model to avoid overfitting. Exact two-sided *P* values were reported where appropriate, whereas values below 0.001 were reported as *P* < 0.001. No formal adjustment for multiple comparisons was applied to the secondary nutritional, inflammatory, and cellular immune outcomes; therefore, these analyses were considered exploratory. A two-sided *P* value <0.05 was considered statistically significant.

## 3 Results

### 3.1 Baseline and perioperative characteristics

After PSM, 40 matched pairs were retained. Six patients in the observation group and eight patients in the control group could not be matched within the prespecified caliper and were therefore excluded from the matched analysis. Although conventional significance testing did not identify statistically significant differences in the measured baseline characteristics before matching, several covariates showed absolute SMDs >0.10, including disease classification, surgical approach, preoperative albumin, and preoperative antibiotic use time. Among the covariates included in the propensity score model, the maximum absolute SMD decreased from 0.227 before matching to 0.079 after matching. After matching, all included baseline covariates had absolute SMDs <0.10, indicating acceptable covariate balance between the two groups. Specifically, after matching, laparoscopic-assisted pull-through was performed in 25 patients (62.5%) in the observation group and 24 patients (60.0%) in the control group, whereas transanal pull-through was performed in 15 (37.5%) and 16 (40.0%) patients, respectively; the surgical approach was well balanced between groups (SMD=0.051, *P* = 0.818). Operative time and intraoperative blood loss were also comparable between the matched groups. Detailed pre- and post-matching balance diagnostics are presented in Table 1.

**Table 1:** Comparison of baseline and perioperative characteristics between the two groups before and after propensity score matching.

Variable	Observation group before matching (*n* = 46)	Control group before matching (*n* = 48)	SMD	*P* value	Observation group after matching (*n* = 40)	Control group after matching (*n* = 40)	SMD	*P* value
Age (months)	12.3 ± 6.1	12.8 ± 6.4	0.080	0.699	12.5 ± 5.9	12.8 ± 6.1	0.050	0.824
Gender			0.078	0.706			0.051	0.818
Male	27 (58.7)	30 (62.5)			24 (60.0)	25 (62.5)		
Female	19 (41.3)	18 (37.5)			16 (40.0)	15 (37.5)		
Weight (kg)	8.6 ± 1.8	8.5 ± 1.9	0.054	0.794	8.7 ± 1.7	8.6 ± 1.8	0.057	0.799
Disease classification			0.114	0.855			0.079	0.938
Short-segment type	5 (10.9)	6 (12.5)			4 (10.0)	5 (12.5)		
Common type	34 (73.9)	33 (68.8)			30 (75.0)	29 (72.5)		
Long-segment type	7 (15.2)	9 (18.8)			6 (15.0)	6 (15.0)		
Surgical approach			0.227	0.275			0.051	0.818
Laparoscopic-assisted pull-through	30 (65.2)	26 (54.2)			25 (62.5)	24 (60.0)		
Transanal pull-through	16 (34.8)	22 (45.8)			15 (37.5)	16 (40.0)		
Operative time (min)	118.6 ± 22.4	124.1 ± 24.0	0.237	0.254	121.3 ± 22.1	122.6 ± 22.8	0.058	0.796
Intraoperative blood loss (mL)	32.8 ± 14.5	36.9 ± 15.8	0.270	0.194	34.1 ± 14.2	34.8 ± 14.6	0.049	0.828
Preoperative albumin (g/L)	38.2 ± 3.5	37.6 ± 3.8	0.164	0.428	38.0 ± 3.4	37.9 ± 3.6	0.029	0.899
Preoperative antibiotic use time (h)	2.5 ± 0.8	2.6 ± 0.9	0.117	0.571	2.5 ± 0.8	2.5 ± 0.8	0.000	1.000

Continuous variables are presented as mean ± standard deviation, and categorical variables as *n* (%). SMD, standardized mean difference. Propensity score matching was performed using 1:1 nearest-neighbor matching without replacement, with a caliper width of 0.02 on the propensity-score scale. An absolute SMD <0.10 was considered indicative of acceptable covariate balance. For categorical variables with more than two categories, the reported SMD represents the maximum absolute standardized difference across categories. Operative time and intraoperative blood loss were additionally reported as measured indicators of surgical complexity and were not included as covariates in the propensity score model. *P* values are provided for descriptive purposes and were not used as the primary criterion for assessing covariate balance.

### 3.2 Clinical indicators

The observation group had shorter postoperative recovery time of bowel sounds, time to first defecation, and postoperative hospital stay than the control group. The corresponding between-group mean differences were −12.10 h (95% CI: −15.97 to −8.23), −13.30 h (95% CI: −18.36 to −8.24), and −3.90 d (95% CI: −5.53 to −2.27), respectively (all *P* < 0.001). See Table 2.

**Table 2:** Comparison of postoperative intestinal function recovery and hospitalization duration between the two groups.

Indicator	Observation group (*n* = 40)	Control group (*n* = 40)	*t*	*P*
Recovery time of bowel sounds (h)	22.5 ± 7.8	34.6 ± 9.5	−6.24	<0.001
First defecation time (h)	38.5 ± 10.1	51.8 ± 12.5	−5.24	<0.001
Postoperative hospitalization days (d)	10.5 ± 3.0	14.4 ± 4.2	−4.78	<0.001

Among the 40 matched patients in the observation group, 38 (95.0%) met the predefined adherence criterion of receiving at least 80% of the prescribed probiotic and glutamine doses during the 30-day intervention period. The remaining two patients had intermittent missed doses documented during outpatient treatment but completed the planned treatment period without permanent discontinuation. No allergic reactions, serious gastrointestinal intolerance, or other serious adverse events judged to be related to probiotics or glutamine were documented. No patient permanently discontinued either study medication because of an intervention-related adverse event.

### 3.3 Postoperative HAEC

During the 6-month postoperative follow-up, at least one confirmed HAEC episode occurred in 4 of 40 patients in the observation group (10.0%) and 13 of 40 patients in the control group (32.5%) (*χ*^2^ = 6.05, *P* = 0.014). In the post-matching univariable Logistic regression analysis, membership in the observation group was associated with lower odds of HAEC compared with the control group (OR = 0.23, 95% CI: 0.07–0.79, *P* = 0.019). Because only 17 HAEC events occurred in the matched cohort, no additional covariates were entered into this model. For the patient-level incidence analysis, patients with recurrent confirmed HAEC episodes were counted once. See Table 3.

**Table 3:** Comparison of the 6-month postoperative incidence of HAEC between the two groups.

Outcome	Observation group (*n* = 40)	Control group (*n* = 40)	*χ^2^*	*P*
HAEC occurred	4（10.0）	13（32.5）		
No HAEC	36（90.0）	27（67.5）	6.05	0.014

HAEC, Hirschsprung disease-associated enterocolitis. Patients with at least one confirmed HAEC episode during the 6-month follow-up were classified as having HAEC. Recurrent confirmed episodes in the same patient did not increase the patient-level event count.

### 3.4 Nutritional indicators

At baseline, there were no statistically significant between-group differences in serum albumin, prealbumin, or transferrin levels (all *P* > 0.05). At 3 months after surgery, all three nutritional indicators were higher in the observation group than in the control group. For the change-from-baseline analysis, the between-group mean differences were 4.20 g/L for albumin (95% CI: 3.06–5.34), 31.60 mg/L for prealbumin (95% CI: 23.14–40.06), and 0.29 g/L for transferrin (95% CI: 0.17–0.41), respectively (all *P* < 0.001). See Table 4.

**Table 4:** Comparison of baseline, 3-month, and change-from-baseline nutritional indicators between the two groups.

Indicator	Time point	Observation group (*n* = 40)	Control group (*n* = 40)	*t*	*P*
Albumin (g/L)	Baseline	38.0 ± 3.4	37.9 ± 3.6	0.13	0.899
	3 months	38.5 ± 3.0	34.2 ± 3.2	6.20	<0.001
	*Δ* change	0.5 ± 2.4	−3.7 ± 2.7	7.35	<0.001
Prealbumin (mg/L)	Baseline	176.4 ± 21.8	175.1 ± 22.2	0.26	0.792
	3 months	215.6 ± 21.4	182.7 ± 20.3	7.05	<0.001
	*Δ* change	39.2 ± 18.6	7.6 ± 19.4	7.44	<0.001
Transferrin (g/L)	Baseline	2.21 ± 0.31	2.16 ± 0.34	0.69	0.494
	3 months	2.55 ± 0.32	2.21 ± 0.36	4.46	<0.001
	*Δ* change	0.34 ± 0.25	0.05 ± 0.27	4.98	<0.001

Data are presented as mean ± standard deviation. Baseline was defined as the most recent measurement obtained within 48 h before surgery. *Δ* change was calculated as the 3-month value minus the baseline value.

### 3.5 Inflammatory factors

Baseline IL-10 and IL-6 levels were comparable between the two groups (both *P* > 0.05). At 3 months, IL-10 was higher and IL-6 was lower in the observation group than in the control group. For the change-from-baseline analysis, the between-group mean difference was 15.00 pg/mL for IL-10 (95% CI: 13.11–16.89) and −4.90 pg/mL for IL-6 (95% CI: −6.55 to −3.25) (both *P* < 0.001). See Table 5.

**Table 5:** Comparison of baseline, 3-month, and change-from-baseline inflammatory indicators between the two groups.

Indicator	Time point	Observation group (*n* = 40)	Control group (*n* = 40)	*t*	*P*
IL-10 (pg/mL)	Baseline	28.82 ± 3.67	28.51 ± 3.73	0.37	0.709
	3 months	44.32 ± 3.86	29.01 ± 3.54	18.49	<0.001
	*Δ* change	15.50 ± 4.40	0.50 ± 4.10	15.77	<0.001
IL-6 (pg/mL)	Baseline	31.34 ± 3.24	31.11 ± 3.31	0.31	0.754
	3 months	25.54 ± 2.17	30.21 ± 3.17	−7.69	<0.001
	*Δ* change	−5.80 ± 3.60	−0.90 ± 3.80	−5.92	<0.001

Data are presented as mean ± standard deviation. *Δ* change was calculated as the 3-month value minus the baseline value; therefore, a negative *Δ* for IL-6 indicates a decrease from baseline.

### 3.6 Cellular immunity

At baseline, the CD4 percentage and CD4/CD8 ratio did not differ significantly between the two groups (both *P* > 0.05). At 3 months, both indicators were higher in the observation group than in the control group. For the change-from-baseline analysis, the between-group mean difference was 5.40 percentage points for CD4 percentage (95% CI: 3.24–7.56) and 0.31 for the CD4/CD8 ratio (95% CI: 0.20–0.42) (both *P* < 0.001). See Table 6.

**Table 6:** Comparison of baseline, 3-month, and change-from-baseline cellular immune indicators between the two groups.

Indicator	Time point	Observation group (*n* = 40)	Control group (*n* = 40)	*t*	*P*
CD4 (%)	Baseline	36.4 ± 6.0	36.1 ± 6.1	0.22	0.825
	3 months	42.1 ± 5.9	36.4 ± 6.2	4.21	<0.001
	*Δ* change	5.7 ± 4.8	0.3 ± 4.9	4.98	<0.001
CD4/CD8	Baseline	1.29 ± 0.31	1.28 ± 0.32	0.14	0.887
	3 months	1.62 ± 0.33	1.30 ± 0.30	4.54	<0.001
	*Δ* change	0.33 ± 0.24	0.02 ± 0.25	5.66	<0.001

Data are presented as mean ± standard deviation. *Δ* change was calculated as the 3-month value minus the baseline value.

## 4 Discussion

Although radical surgery for Hirschsprung disease relieves the mechanical obstruction of the distal bowel, postoperative bowel recovery may also be influenced by mucosal healing, nutritional status, inflammatory responses, and other perioperative factors (11, 12). Based on a single-center retrospective observational cohort, this study evaluated postoperative outcomes associated with a 1-month combined regimen of probiotics and glutamine in children with HD. More favorable postoperative bowel recovery, HAEC incidence, and nutritional and immune indicators were observed in the observation group than in the control group. However, because no probiotic-only or glutamine-only group was included, the individual contribution of each component and any potential interaction between the two interventions cannot be determined from the present study.

The recovery of intestinal motility in the early postoperative period directly affects the length of hospital stay and medical burden. In the observation group, the recovery time of bowel sounds was shortened by an average of about 12.1 h, the first defecation was 13.3 h earlier, and the postoperative hospitalization days were reduced by nearly 4 days. Notably, operative time and intraoperative blood loss were also well balanced between the two groups after matching, with absolute SMDs of 0.058 and 0.049, respectively. These findings make a major measured between-group difference in surgical complexity less apparent as an explanation for the observed difference in postoperative bowel recovery, although subtle intraoperative factors such as pelvic nerve injury cannot be completely excluded in a retrospective study. Previous studies suggest that glutamine may support intestinal epithelial metabolism and barrier integrity, whereas probiotics may influence gut microbial composition and host–microbe interactions (13–17). These previously proposed mechanisms may provide biological plausibility for the observed differences in postoperative bowel recovery. However, the present study did not directly assess microbiome composition, short-chain fatty acids, intestinal permeability, tight-junction proteins, serotonergic signaling, or probiotic colonization. Therefore, these mechanisms should not be interpreted as pathways directly demonstrated by the present data.

During the 6-month postoperative follow-up, HAEC occurred in 10.0% of patients in the observation group and 32.5% in the control group, corresponding to an absolute between-group difference of 22.5 percentage points. The observed difference in HAEC incidence may be biologically related to mechanisms involving the intestinal barrier, gut microbiota, and immune regulation. Previous studies suggest that glutamine may support intestinal barrier integrity, whereas probiotics may influence microbial composition and mucosal immune regulation (18–20). These mechanisms provide biological plausibility for the observed association, but microbiome composition, intestinal permeability, and mucosal immune markers were not directly measured in the present study. In the present retrospective study, HAEC events were adjudicated using predefined clinical, radiographic, and laboratory criteria rather than a routinely documented numerical HAEC score. Independent review with treatment-group information masked was used to reduce outcome-assessment bias; nevertheless, some diagnostic misclassification, particularly for mild outpatient episodes with limited objective documentation, cannot be completely excluded. The present findings support further evaluation of this combined regimen; however, direct superiority over either component alone or a synergistic interaction between probiotics and glutamine cannot be inferred from the current study design.

Baseline albumin, prealbumin, and transferrin levels were comparable between the two matched groups, whereas change-from-baseline analysis showed greater increases in all three indicators in the observation group by 3 months. These findings indicate a more favorable change in the measured nutritional profile in the observation group, although the retrospective observational design does not establish that the combined intervention directly caused these differences. Previous studies have suggested that glutamine may participate in energy metabolism and provide substrates for intestinal epithelial and immune-cell metabolism, which may offer a biologically plausible explanation for the observed association (21, 22). In particular, the relatively short half-life of prealbumin makes it responsive to recent changes in nutritional status; therefore, its higher level at the 3-month assessment may reflect a more favorable nutritional status after the intervention period. However, because postoperative nutrition, growth, disease severity, and other nutritional supplementation may also influence these markers, the contribution of probiotics combined with glutamine cannot be isolated from the present data.

Baseline CD4 percentage and CD4/CD8 ratio were comparable between the two groups, whereas both indicators showed greater increases from baseline in the observation group at 3 months. These findings indicate between-group differences in the trajectories of the measured cellular immune indicators. One possible biological explanation involves the gut-immune axis. Previous studies have suggested that intestinal barrier integrity and microbial metabolites may influence systemic immune regulation and T-cell responses (23–26). Such mechanisms may provide biological plausibility for the observed differences; however, intestinal barrier function, microbial metabolites, regulatory T-cell activity, and related immune pathways were not directly measured in the present study. Furthermore, although the differences in CD4 proportion and CD4/CD8 ratio were observed at 3 months, the present data do not establish persistent immune remodeling or an “immune imprinting” effect.

The changes in inflammatory markers may provide additional context for the observed clinical associations. Baseline IL-10 and IL-6 levels were comparable between the groups, whereas the observation group showed a greater increase in IL-10 and a greater decrease in IL-6 from baseline to 3 months. Previous studies have suggested that glutamine and probiotics may influence inflammatory signaling through effects involving intestinal barrier function, microbial metabolites, and immune regulation (27, 28). These previously proposed pathways may partly explain the biological plausibility of the observed differences in IL-6 and IL-10. However, microbial metabolites, intestinal permeability, NF-*κ*B signaling, dendritic-cell function, and Th1/Th17 or Treg-related responses were not directly evaluated in this study. Therefore, the present findings should be interpreted as differences in measured circulating inflammatory markers rather than evidence that the combined regimen directly induced specific anti-inflammatory pathways or immune remodeling.

Several limitations should be considered. First, as a single-center retrospective observational cohort, this study remains susceptible to residual and unmeasured confounding despite propensity score matching. PSM can improve balance only for measured covariates included in the model and cannot eliminate potential indication bias or residual differences related to physician treatment preference, prior HAEC history, aspects of disease severity not fully captured by segment classification, surgeon- or team-related factors, detailed postoperative antibiotic regimens, intercurrent infection, nutritional supplementation, growth trajectories, perioperative management, or family adherence. In addition, no formal sensitivity analyses using alternative propensity-score specifications, caliper widths, or analytic approaches were performed; therefore, the robustness of the findings to alternative propensity-score methods could not be evaluated. No formal adjustment for multiple comparisons was applied to the secondary nutritional, inflammatory, and cellular immune outcomes; therefore, these analyses should be considered exploratory, and the potential for an increased type I error should be taken into account when interpreting the results. Therefore, the observed associations should not be interpreted as demonstrating a causal treatment effect. Second, the relatively small sample size and exclusion of patients with total colonic aganglionosis and exclusion of patients with total colonic aganglionosis limited the ability to perform reliable subgroup analyses and may reduce the generalizability of the findings to the full spectrum of HD. Third, the 6-month follow-up period did not permit evaluation of longer-term bowel function, growth and development, or quality of life. In addition, HAEC ascertainment relied on retrospective clinical documentation rather than a prospectively applied numerical scoring system; therefore, mild episodes managed outside our institution or episodes with incomplete objective documentation may have been under-ascertained. Fourth, microbiome data, intestinal barrier markers, and other mechanistic measurements were not collected, preventing direct verification of the biological mechanisms discussed above. Finally, because probiotics and glutamine were administered as a fixed combination and the control group received neither study intervention, the present study cannot distinguish the independent contribution of probiotics from that of glutamine or determine whether the two components have an interactive effect. Future multicenter prospective studies with longer follow-up and, ideally, randomized treatment allocation are needed to confirm these associations and more accurately determine the potential role of this combined regimen in postoperative management.

## Data Availability

The raw data supporting the conclusions of this article will be made available by the authors, without undue reservation.
